# Kampo Medicine for Various Aging-Related Symptoms: A Review of Geriatric Syndrome

**DOI:** 10.3389/fnut.2020.00086

**Published:** 2020-07-15

**Authors:** Shin Takayama, Naoki Tomita, Ryutaro Arita, Rie Ono, Akiko Kikuchi, Tadashi Ishii

**Affiliations:** ^1^Department of Kampo and Integrative Medicine, Tohoku University Graduate School of Medicine, Sendai, Japan; ^2^Department of Education and Support for Regional Medicine, Tohoku University Hospital, Sendai, Japan; ^3^Department of Kampo Medicine, Tohoku University Hospital, Sendai, Japan; ^4^Department of Geriatrics and Gerontology, Institue of Development, Aging and Cancer, Tohoku University, Sendai, Japan

**Keywords:** aging, Kampo medicine, geriatric syndrome, elderly, evidence

## Abstract

With the continued growth of the aging population in Japan, geriatric syndrome (GS), which is associated with aging-related symptoms, has become a social problem. GS is caused by physiological and pathological aging and may manifest various symptoms. Physicians use multidisciplinary approaches to provide treatment for individual GS symptoms. Kampo medicine, a Japanese traditional medicine that uses multiple pharmacologically active substances, is useful for many syndromes, conditions, disorders, and diseases associated with GS. Evidence of the effectiveness of Kampo medicine for GS has accumulated in recent years. The effects of Kampo treatment for symptoms related to functional decline of the cardiovascular, respiratory, and digestive systems, cognitive impairment and related disorders, pain and other sensory issues, among others, support the use of Kampo medicine for the management of GS. The role of Kampo medicine for GS is summarized in this review.

## Introduction

Geriatric syndrome (GS) is a well-known clinical entity characterized by symptoms highly prevalent in old age. It presents with multiple contributing factors, including physiological aging, and requires a multidisciplinary approach. When compared to the “disease” entity, the differences in features are unknown etiology and inclusion of physiological aging as a cause (1). There is no universal definition of GS; there are some variations in the features included in GS definitions. These variations in definition lead to ambiguity regarding the included symptoms. Originally, GS symptoms were expressed as the 3Ms (mentality, micturition, and mobility) or 4Is (immobility, instability, impaired cognition, and incontinence—also named the “geriatric giants”) (2). Today, 20 or more symptoms are listed (3). The majority of GS symptoms emerge slowly and are chronic, with a low risk of mortality by themselves. Consequently, they tend to be overlooked as physiological changes, resulting in increased dependency. GS is clinically significant as a warning sign for the risk of increased care dependency. When determining countermeasures for GS, we must exclude the possibility of a single cause before considering multifactorial etiology (3, 4) (e.g., endocrine disorders) (5). Then, we must exclude medication-related side effects (6).

Kampo medicine is effective in many cases of GS with multiple causes. Original GS symptoms (such as symptoms related to functional decline of the cardiovascular, respiratory, and digestive systems; cognitive impairment and related disorders; and pain and other sensory issues) are considered treatment targets. Because of the multifactorial nature of GS and specialization in medicine, care for those with GS tends to be fragmented. Kampo medicine could prevent fragmentation of patient care.

On the other hand, medical expenses amounted to more than one third of social security expenses in 2018 (7). With the growing “super-aging” society and the declining birth rate in Japan, medical expenses are only expected to increase. In light of these points, we have herein summarized the efficacy, safety, and social economic advantage associated with the use of Kampo medicine for GS.

In this review, we summarize randomized controlled trials (RCTs) for GS. When no RCT was available for specific conditions or disorders, some observational studies were described. Details of each Kampo medication are listed on the Standards of Reporting Kampo Products (STORK) website (http://mpdb.nibiohn.go.jp/stork/) (8). The names of Kampo medicines were abbreviated according to the Japan Society of Oriental Medicine (9).

## Kampo Medicine for GS

### Kampo Medicine for Cardiovascular Disease and Related Symptoms

Generally, cardiovascular disease and related disorders increase with age. The Japanese lifestyle has shifted toward a Western lifestyle; thus, cardiovascular disease has increased in the last half century. Western medications are commonly used to control hypertension and related diseases; furthermore, they have been shown to effectively suppress cardiovascular events. A few clinical trials have been conducted on Kampo medicine for the treatment of cardiovascular disease and related symptoms. Overall, these RCTs were conducted to manage symptoms difficult to control in Western medicine. Soft endpoints were the improvement of accessory symptoms of hypertension, orthopedic hypotension related to diabetes mellitus, and edema according to deep vein thrombosis of the lower limb (Table 1).

**Table 1:** RCT of Kampo medicine in cardiovascular and related conditions.

**References**	**Study design**	**Subjects (*n*)**	**Age, years (mean ± SD)**	**Disease/symptom**	**Kampo formulation**	**Comparator**	**Outcome**	**Adverse reaction**
Uchida (12)	RCT	12	65.8 ± 16.3	Acute symptomatic proximal deep vein thrombosis	KBG added to conventional treatment	Conventional treatment with thrombolysis and anticoagulant	The improvement rates of circumference difference between the affected and the normal limbs were significantly higher in the KBG group.	N/A
Arakawa et al. (10)	DB-RCT	204	52.3 ± 11	Accessory symptoms of hypertension	OGT	Placebo	Efficacy was significantly higher in the OGT group based on the total score for the accessory symptoms of hypertension, especially hot flushes and facial suffusion.	None
Nakamura et al. (11)	Crossover RCT	10	57.6 ± 8.1	Orthopedic hypotension related with diabetes mellitus	GRS	Placebo	The change of orthopedic systolic and diastolic pressure was significantly lower in the GRS group than in the placebo group. All patients complained of dizziness in the placebo group, but only 10% complained of the symptom in the GRS group.	N/A

*DB, double-blinded; GRS, goreisan; KBG, keishibukuryogan; OGT, orengedokuto; RCT, randomized controlled trial; SD, standard deviation; N/A, not assigned*.

#### Hypertension

Arakawa et al. conducted a double-blinded (DB) RCT on orengedokuto (OGT) for the treatment of accessory symptoms of hypertension; the study included elderly subjects (10). Efficacy was significantly higher in the OGT group based on the total score for the accessory symptoms of hypertension; sub-analysis showed the efficacy to be higher for hot flashes and facial suffusion in the OGT group. However, there were no significant differences between the OGT and placebo groups regarding the decrease of blood pressure or the antihypertensive effect.

#### Hypotension

Nakamura et al. reported the efficacy of goreisan (GRS) for orthopedic hypotension related with diabetes mellitus in an RCT that included elderly subjects (11). The change in orthopedic systolic and diastolic pressure was significantly lower in the GRS group than in the placebo group. All patients complained of dizziness in the placebo group, but only 10% complained of the symptom in the GRS group.

#### Disorders Related to Vein Dysfunction

Uchida reported the effect of keishibukuryogan (KBG) for edema according to deep vein thrombosis of the lower limb in an RCT of elderly subjects (12). The improvement rates of circumference difference between the affected and the normal limbs were significantly higher in the KBG group than in the conventional treatment group.

### Kampo Medicine for Aspiration Pneumonia and Chronic Obstructive Pulmonary Disease

Respiratory disease is increasing with the aging of society. Kampo medicine has been effective at treating acute respiratory infection, and there are some reports that Kampo medicine has a prophylactic effect in aspiration pneumonia and acute exacerbation of chronic obstructive pulmonary disease (COPD) (Table 2).

**Table 2A:** Studies of Kampo medicine for aspiration pneumonia.

**References**	**Study design**	**Subjects (*n*)**	**Age, years (mean ± SD or range) Kampo group/control group**	**Disease/symptom**	**Kampo formulation**	**Comparator**	**Outcome**	**Adverse reaction**
Iwasaki et al. (15)	Controlled clinical trial	32	76.4 ± 3.1/70.5 ± 5.6	Aspiration pneumonia	HKT	Placebo	The swallowing reflex was significantly improved and substance-P in saliva increased significantly in the Kampo group.	N/A
Iwasaki et al. (17)	RCT	16	79.6 ± 4.3/70.5 ± 5.6	Aspiration pneumonia, lacunar infarction, or brain atrophy	HKT	Placebo	Cough reflex was significantly improved after HKT administration.	N/A
Mantani et al. (24)	RCT	15	78.7 (65–96)/80.5 (72–93)	Aspiration pneumonia	SHT	No Kampo, only conventional therapy	The mean values of fever index, CRP, and antibiotics use were decreased significantly in the SHT group. The latency of the swallowing reflex was not significantly changed.	None
Iwasaki et al. (18)	RCT	95 (92)	84.5 ± 6.8/83.1 ± 7.2	Aspiration pneumonia in dementia	HKT	Placebo	HKT reduced pneumonia onset and tended to reduce pneumonia-related mortality. The relative risk of pneumonia in the Kampo group compared with the control group was 0.51, and that of death from pneumonia was 0.41.	None
Kawago et al. (19)	DB-RCT (envelope)	34 (30)	65.2 ± 13.9/69.2 ± 13.0	Aspiration pneumonia after cardiovascular surgery	HKT	Placebo	The rate of postoperative aspiration pneumonia was significantly lower in the Kampo group than in the placebo group. White blood cell counts and CRP levels on postoperative day 3 were significantly lower in the Kampo group.	N/A

*CRP, C-reactive protein; DB, double-blinded; HET, hochuekkito; HKT, hangekobokuto; RCT, randomized controlled trial; SD, standard deviation; SHT, seihaito; N/A, not assigned*.

#### Kampo Medicine for Aspiration Pneumonia

Pneumonia is one of the leading causes of death in the elderly. Therefore, preventing pneumonia, including aspiration pneumonia, is very important. Aspiration pneumonia occurs frequently in patients with cerebrovascular disease, patients with neurodegenerative disease, and bedridden patients with dysphagia and depression of swallowing and cough reflex. Patients with swallowing or coughing impairment have low levels of substance-P in their saliva (13, 14). Substance-P is a neuropeptide that plays an important role in swallowing and cough reflexes (13). Table 2A shows studies of Kampo medicine for aspiration pneumonia.

Iwasaki et al. reported that hangekobokuto (HKT) improves swallowing reflex and increases salivary levels of substance-P in patients who had a stroke (15). They also reported that HKT improves swallowing reflex in patients with Parkinson's disease despite no significant changes in their salivary levels of substance-P (16). Iwasaki et al. also showed that HKT improves cough reflex of patients with cerebral atrophy and lacunar infarction (17), reduces the risk of aspiration pneumonia in the elderly, and maintains self-feeding capacity better than the control (18). Additionally, Kawago et al. reported that HKT prevents aspiration pneumonia in patients after cardiovascular surgery (19). HKT is thought to act via regulation of the cerebral levels of 5-hydroxytryptamine, noradrenaline, and dopamine (20). Impairment of the swallowing reflex correlates strongly with decreased dopamine levels in the basal ganglia (21). Therefore, HKT-induced improvement of swallowing reflex may be associated with HKT-induced increase in brain dopamine levels. Hochuekkito (HET) is another Kampo formula for prevention of aspiration pneumonia. Tamano et al. reported that administration of HET, alone or in combination with rehabilitation, reduces the number of hospitalizations due to aspiration pneumonia (22, 23). HET also improves clinical symptoms such as appetite loss and general malaise, increases body weight and serum albumin, and increases temperature in patients with low body temperature. Mantani et al. reported that seihaito (SHT), added to conventional treatment, decreases the mean values of fever, C-reactive protein (CRP) levels, and antibiotics use compared with conventional therapy alone (24). However, SHT does not improve the latency of swallowing reflex. This study indicated that SHT has an anti-inflammatory effect in patients with recurrent aspiration pneumonia but does not improve swallowing reflex. Iwasaki et al. reported that xanthine oxidase activity in lung tissues is elevated in a mouse model of aspiration pneumonia and that SHT is able to reverse this elevation (25). The authors speculated that SHT pretreatment can reduce oxygen radical production in inflamed lungs. Dysphagia is also considered to relate to gastroesophageal reflux disease (GERD).

#### Kampo Medicine for COPD

COPD does not affect solely the airways; it is considered a systemic inflammation. The treatment guidelines for COPD recommend the use of bronchodilators, inhaled corticosteroids, and rehabilitation. One of the main goals of COPD treatment is to prevent acute exacerbation, which is known to affect patient prognosis. Table 2B shows an RCT of Kampo medicine for COPD.

**Table 2B:** RCT of Kampo medicine for COPD.

**References**	**Study design**	**Subjects (*n*)**	**Age, years (range or mean ± SD) Kampo group/control group**	**Disease/symptom**	**Kampo formulation**	**Comparator**	**Outcome**	**Adverse reaction**
Sasaki et al. (26)	RCT (envelope)	19	62–83/65–88	Chronic respiratory disease	BAK	Bromhexine hydrochloride	The BAK group showed improved loosening of phlegm after 2 and 4 weeks.	None
Kato et al. (28)	RCT (envelope)	31	66.7 ± 7.1/66.7 ± 6.4	COPD	SHT + smoking cessation	No Kampo, only smoking cessation	SHT improved the clinical symptoms of patients with COPD for 6 months, and chest X-ray or CT findings at 24 months.	N/A
Shinozuka et al. (30)	RCT	35	73 ± 1	COPD	HET + bronchodilators	Bronchodilators	In the HET group, serum CRP and TNF-α significantly decreased, and serum albumin level was significantly increased.	N/A
Tatsumi et al. (31)	RCT (envelope)	71	Elderly	COPD	HET + conventional therapy	Conventional therapy	In the HET group, body weight significantly increased for 6 months, and St. George's Respiratory Questionnaire score decreased, indicating that quality of life improved. The number of common cold and acute exacerbations was significantly lower. CRP, TNF-α, and IL-6 decreased, and serum prealbumin increased.	None
Mukaida et al. (27)	Crossover RCT	24 (23)	Group A: 76.2 ± 8.5/group B: 79.2 ± 2.6	COPD	BAK	No Kampo	BAK significantly improved VAS scores for cough frequency in group A. VAS scores for cough intensity in each group tended to improve. BAK improved scores of cough severity significantly.	Serum ALP elevation in two participants

*ALP, alkaline phosphatase; BAK, bakumondoto; COPD, chronic obstructive pulmonary disease; CRP, C-reactive protein; CT, computed tomography; HET, hochuekkito; IL, interleukin; SHT, seihaito; TNF-α, tumor necrosis factor-α; VAS, visual analog scale; N/A, not assigned*.

Among Kampo medications, bakumondoto (BAK) and SHT have been shown to improve the symptoms of COPD. Sasaki et al. reported that BAK significantly helps loosen phlegm of patients with chronic respiratory disease (26). Mukaida et al. showed that BAK significantly improves visual analog scale (VAS) scores for cough frequency, but not for cough intensity (27). Kato et al. reported that administration of SHT improves the clinical symptoms of COPD (28). BAK is thought to exert a peripheral antitussive effect by inhibiting the synthesis or release of nitric oxide (29). According to the traditional theory, BAK should be used for patients with dry cough and SHT for patients with productive cough.

Shinozuka et al. and Tatsumi et al. reported that HET reduces the number of common cold and acute exacerbation episodes in patients with COPD (30, 31). HET decreased serum CRP, tumor necrosis factor (TNF)-α, and interleukin-6 levels and increased serum prealbumin levels. Furthermore, HET resulted in a significant increase in body weight over 6 months and a decrease in St. George's Respiratory Questionnaire score, indicating an improvement in quality of life (QOL). HET has antiviral and anti-inflammatory effects, thus contributing to preventing exacerbation (32). Jo et al. reported that daikenchuto (DKT) reduces exacerbation in patients with COPD (33). Patients treated with DKT had a significantly lower risk of rehospitalization or death after discharge. By improving bowel movements and tolerance to muscarinic antagonists, DKT improves the respiratory status of patients with COPD. Recently, there have been a few DB-RCTs on traditional Chinese medicine for patients with COPD (34–36). In the majority of the studies, crude drugs added to the conventional therapy prevented recurrence of acute exacerbation.

### Kampo Medicine for the Digestive System

Kampo medicine was developed to control and maintain the function of the digestive system. It is used to enhance motility in the gastrointestinal tract and promote digestion. Kampo medicine has recently been used for early recovery from surgical intervention, especially for elderly patients receiving cancer treatment. RCTs have explored the use of Kampo medicine for constipation, perioperative symptoms, and conditions in the gut, functional dyspepsia (FD), GERD, and nonerosive reflux disease (NERD) (Table 3). After development of a placebo of these Kampo medicines, DB-RCTs using DKT or rikkunshito (RKT) were conducted. RCTs showed that DKT can be used for preventing postoperative ileus, improving bowel movement in the early days, improving QOL, having anti-inflammatory effects, improving early oral intake, enhancing total oral/enteral caloric intake and portal venous flow volume, and minimizing weight loss after abdominal surgery in the perioperative stage. RCTs showed that RKT can be used for improving upper gastric symptoms (globus sensation, delayed gastric emptying, abdominal bloating, heavy feeling in the stomach, sick feeling after meals, heartburn after meals, and epigastric pain), psychological symptoms, appetite loss, acyl ghrelin levels, and a low body mass index in FD, GERD, and NERD. Table 3A shows an RCT of Kampo medicine for FD and GERD.

**Table 3A:** RCT of Kampo medicine for FD and GERD.

**References**	**Study design**	**Subjects (*n*)**	**Age, years (mean ± SD or range)**	**Disease/symptom**	**Kampo formulation**	**Comparator**	**Outcome**	**Adverse reaction**
Tominaga et al. (37)	DB-RCT	125	50.4 ± 13.7 (23–76)/50.4 ± 14.9 (23–83)	Functional dyspepsia	RKT	Placebo	RKT increased overall treatment efficacy. RKT improved upper gastrointestinal symptoms, especially postprandial fullness/early satiety and bloating. Improvement of the Hospital Anxiety and Depression Scale correlated with those of the Patient Assessment of Upper Gastrointestinal Disorders-Symptom Severity Index, the Global Overall Symptom scale, and the modified Frequency Scale for the Symptoms of Gastroesophageal Reflux Disease, postprandial fullness/early satiety, dyspepsia, and postprandial distress syndrome. RKT may be beneficial for patients with FD to simultaneously treat gastrointestinal and psychological symptoms.	None
Tominaga et al. (38)	DB-RCT	242	62.1 (25–85)/59.4 (22–83)	Patients with PPI-refractory non-erosive reflux disease	RKT with PPI	Placebo with PPI	The mental component summary scores improved more in RKT administration, especially more effective in patients with a low body mass index and significantly improved the acid-related dysmotility symptoms in female and elderly patients.	Nausea, mild cough, dizziness, diarrhea, etc. (low level)
Sakata et al. (39)	DB-RCT	95	72.1 (65–85)/73.4 (65–83)	Patients with PPI-refractory non-erosive reflux disease	RKT with PPI	Placebo with PPI	The degree of improvement of total and acid-related dysmotility symptoms scores was significantly greater in the RKT group. Combination therapy with RKT showed significant improvement in abdominal bloating, heavy feeling in stomach, sick feeling after meals, and heartburn after meals.	N/A
Suzuki et al. (41)	DB- RCT	247	54.5 ± 16.2 (22–85)/53.6 ± 16.0 (21–85)	Functional dyspepsia	RKT	Placebo	Administration of RKT reduced dyspepsia, particularly symptoms of epigastric pain and postprandial fullness. Among *Helicobacter pylori*-positive individuals, RKT improved acyl ghrelin levels.	None
Hayakawa et al. (42)	DB- RCT	23	75 ± 11/70 ± 13	Patients who were projected to require intragastric tube feeding	RKT	Metoclopramide	RKT group reached 50% of the target amount of enteral feeding significantly earlier than the metoclopramide group. RKT increased the plasma level of active ghrelin.	N/A
Tokashiki et al. (43)	RCT	22	55.9 (39–76)/56.6 (25–76)	PPI-refractory laryngopharyngeal reflux	RKT	RKT with PPI	RKT significantly decreased the globus sensation. It also improved delayed gastric emptying. A significant positive correlation between improvements in globus sensation and in gastric emptying were shown.	N/A
Tominaga et al. (44)	DB-RCT	101	63.6 (25–86)/64.5 (25–90)	Refractory GERD with PPI	RKT with a standard dose of PPI	Double dose of PPI	RKT addition significantly decreased the frequency scale of the GERD symptom score similar to treatment with a double dose of PPI.	None
Arai et al. (45)	RCT	27	56.5 ± 15.0/59.0 ± 14.0	FD	RKT	Domperidone	A significant improvement was shown in dyspeptic symptoms treated with either RKT or domperidone. RCT increased acylated ghrelin. The symptom improvement of reflex and ingestion were well correlated with the increase of acylated ghrelin.	N/A
Kato et al. (40)	RCT	19	74.1 ± 6.4/71.7 ± 5.2	GERD with cough, sputum, pharyngolaryngeal discomfort, or mild dyspnea	HKT	no HKT	HKT significantly improved respiratory symptoms related with GERD.	N/A
Harasawa et al. (46)	DB-RCT	235	53.5 (23–79)/52.3 (21–78)	Dysmotility-like dyspepsia	Regular dose of RKT	Low dose of RKT	Regular dose of RKT improved dysmotility-like dyspepsia.	Gastrointestinal symptom, liver dysfunction, or pseudo aldosteronism were shown, the frequency and severity were low
Tatsuta et al. (47)	RCT	42		Chronic dyspepsia	RKT	Placebo	Gastric emptying was significantly accelerated and gastrointestinal symptoms were significantly reduced in patients treated with RKT.	N/A
Miyoshi et al. (48)	RCT	246	Under 20 to over 80 years	Non-ulcer dyspepsia	RKT	Cisapride	RKT significantly improved symptom of appetite loss, epigastric pain, abdominal discomfort, cold feeling of limb, dazzling compared with cisapride. In subanalysis, RKT was more effective in patients over 60 years old, thin, and with water retention.	N/A

*HKT, hangekobokuto; PPI, proton-pump inhibitor; RKT, rikkunshito; N/A, not assigned*.

#### FD and GERD

Tominaga et al. reported the effects of RKT administration on FD and its correlation with anxiety (37). RKT increased the overall treatment efficacy and improved upper gastrointestinal symptoms, especially postprandial fullness/early satiety and bloating. Improvement of the Hospital Anxiety and Depression Scale was correlated with that of the Patient Assessment of Upper Gastrointestinal Disorders–Symptom Severity Index, the Global Overall Symptom scale, and the modified Frequency Scale for the Symptoms of Gastroesophageal Reflux Disease (postprandial fullness/early satiety, dyspepsia, and postprandial distress syndrome). This suggests that RKT may be beneficial for patients with FD to simultaneously treat gastrointestinal and psychological symptoms. Tominaga et al. also studied the use of RKT for patients with NERD refractory to proton-pump inhibitors (PPIs) in a DB-RCT that included elderly subjects (38). The mental component summary scores improved more in the RKT group than in the control group, especially in patients with a low body mass index, and significantly improved the acid-related dysmotility symptoms in female and elderly patients. Sakata et al. reported additional analysis of this study (39). Especially in the elderly, the degrees of improvement in the total and acid-related dysmotility symptom scores were significantly greater in the RKT group. Combination therapy with RKT led to significant improvement in abdominal bloating, heavy feeling in the stomach, sick feeling after meals, and heartburn after meals. Kato et al. reported that HKT significantly improved respiratory symptoms associated with GERD (40). Suzuki et al. reported the efficacy and safety of RKT for FD in a DB-RCT that included elderly subjects (41). Administration of RKT reduced dyspepsia, particularly the symptoms of epigastric pain and postprandial fullness. Among the patients positive for *Helicobacter pylori*, RKT improved acyl ghrelin levels. In a DB-RCT, Hayakawa et al. reported the effects of RKT on enteral feeding and plasma ghrelin levels in critically ill elderly patients (42). The RKT group reached 50% of the target amount of enteral feeding significantly earlier than the metoclopramide group. RKT increased the plasma level of active ghrelin. Tokashiki et al. reported the effects of RKT on the globus sensation in patients with PPI-refractory laryngopharyngeal reflux in an RCT that included elderly subjects (43). RKT or RKT with PPI significantly decreased the globus sensation and improved delayed gastric emptying. A significant positive correlation was found between improvements in the globus sensation and gastric emptying. Tominaga et al. reported the efficacy of RKT for patients with GERD refractory to treatment with PPI in a DB-RCT that included patients from 20 to 90 years of age (44). RKT combined with PPI significantly decreased the frequency scale of the GERD symptoms' score, similar to the decrease seen after treatment with a double dose of PPI. Subgroup analysis showed that the improvement rate of male NERD patients in the RKT group was significantly greater. Subgroup analysis also showed that the patients of male sex or with a low body mass index showed more improvement than other subgroups. Furthermore, no adverse events were observed in this study. Additional RKT therapy for patients with GERD refractory to PPI treatment seemed to be more effective for NERD, male, or low-body mass index patients, and the therapy was shown to be safe. Arai et al. reported a significant improvement in dyspeptic symptoms in patients treated with either RKT or domperidone (45). The improvements of reflux and indigestion symptoms in patients treated with RKT showed good correlations with the increased levels of acylated ghrelin. Harasawa et al. conducted a DB-RCT on RKT for the treatment of dysmotility-like dyspepsia in elderly subjects (46). The regular dose of RKT improved dysmotility-like dyspepsia significantly more than the low dose of RKT. Tatsuta et al. reported the effects of RKT on gastric emptying and gastrointestinal symptoms in dyspeptic patients (47). Gastric emptying was significantly accelerated, and gastrointestinal symptoms were significantly reduced in patients treated with RKT. Miyoshi et al. examined the effects of RKT for complaints related to gastrointestinal function in an RCT that included elderly subjects (48). RKT significantly improved the symptoms of appetite loss, epigastric pain, abdominal discomfort, cold feelings of the limb, and dazzling when compared with cisapride. In a subanalysis, RKT was more effective in patients over 60 years of age, with a thin type body, and with water retention.

#### Constipation

Table 3B shows an RCT of Kampo medicine for constipation. Numata et al. reported the efficacy of DKT for functional constipation in elderly patients after stroke (49). Constipation scoring system points, especially the frequency of bowel movements, feeling of incomplete evacuation, and need for an enema/disimpaction, improved significantly with the addition of DKT. The gas volume score also significantly reduced with the addition of DKT. Arita et al. performed a responder analysis of DKT treatment for constipation in poststroke patients (50). The total neurogenic bowel dysfunction score and Gastrointestinal Symptom Rating Scale (GSRS)-constipation subscale score were significantly reduced after DKT administration. The total neurogenic bowel dysfunction score, GSRS-constipation subscale score, and gas volume score at baseline were significantly correlated with the change in these scores, suggesting that higher scores in these categories and a higher gas volume in the gut may be predictors of response to DKT. Horiuchi et al. reported the effect of DKT in patients with chronic constipation in an RCT that included elderly subjects (51). The addition of DKT to sennoside resulted in a significant improvement in bloating and abdominal pain and a significant decrease in the gas volume score comparing a regular dose and a half dose of DKT. Miyoshi et al. reported the effect of daiokanzoto (DKZT) in patients with chronic constipation in an RCT that included elderly subjects (52). DKZT was significantly more effective for constipation than the placebo. A regular dose of DKZT has a strong effect on some patients; as such, the dose should be determined considering the patient's condition.

**Table 3B:** RCT of Kampo medicine for constipation.

**References**	**Study design**	**Subjects (*n*)**	**Age, years (mean ± SD)**	**Disease/symptom**	**Kampo formulation**	**Comparator**	**Outcome**	**Adverse reaction**
Arita et al. (50)	RCT subanalysis	34	77.5 ± 11.9 78.7 ± 12.1	Functional constipation	DKT added to conventional therapy	Conventional therapy	The total neurogenic bowel dysfunction score, Gastrointestinal Symptom Rating Scale-constipation subscale score, and gas volume score at baseline were significantly correlated with the change in these scores.	N/A
Numata et al. (49)	RCT	34	78.1 ± 11.6	Functional constipation	DKT added to conventional therapy	Conventional therapy	The frequency of bowel movements, feeling of incomplete evacuation, and need for enema/disimpaction were significantly improved by DKT. The gas volume score was also significantly reduced by DKT.	Liver dysfunction (low level)
Horiuchi et al. (51)	RCT	22	69.2 ± 13/68.9 ± 16	Chronic constipation	Regular dose of DKT added to sennoside	Half of regular dose of DKT added to sennoside	The addition of DKT reduced abdominal bloating and pain in chronic constipation patients receiving stimulant laxatives with decreasing the bowel gas volume.	None
Miyoshi et al. (52)	DB-RCT	146	65 patients over 60 years	Constipation	Regular dose of DKZT	Low dose of DKZT or placebo	DKZT was significantly effective for constipation compared to the placebo.	No significant difference between groups.

*DKT, daikenchuto; DKZT, daiokanzoto; N/A, not assigned*.

#### Perioperative Symptoms and Conditions in the Gut

Table 3C shows an RCT of Kampo medicine for the perioperative period. Nishino et al. reported the effects of DKT after esophageal cancer resection in an RCT that included elderly subjects (53). The rate of weight loss at postoperative day 21 was significantly suppressed in the DKT group. Postoperative bowel symptoms tended to be rare, and the serum CRP level at postoperative day 3 tended to be lower in the DKT group. This suggests that DKT treatment after esophageal cancer resection may promote the recovery of gastrointestinal motility and minimize weight loss; it may also suppress the excess inflammatory reaction related to surgery. In a DB-RCT, Katsuno et al. reported the effect of DKT on elderly patients with colon cancer undergoing open surgery by transit analysis using radiopaque markers (54). The number of radiopaque markers in the anal side of the small intestine at 6 h was significantly greater in the DKT group. This suggests that DKT may contribute to early oral intake in the postoperative course. Okada et al. examined the efficacy of DKT for the prevention of paralytic ileus after pancreaticoduodenectomy in a DB-RCT of elderly patients (55). Perioperative treatment with DKT neither decreased the incidence of clinically relevant postoperative paralytic ileus nor shortened the time to the first postoperative flatus, suggesting that DKT may preclude the routine use of DKT in clinical practice after this operation. Akamatsu et al. reported the effects of DKT on intestinal motility after total gastrectomy in an RCT that included elderly subjects (56). DKT significantly improved the number of stools per day, stool consistency, and gas volume scores. This suggests that DKT promoted early postoperative bowel functions after total gastrectomy. In a DB-RCT, Katsuno et al. reported the clinical efficacy of DKT for gastrointestinal dysfunction following colon surgery in elderly patients (57). Bowel movement frequency in the DKT group at postoperative day 8 was significantly lower than that in the placebo group, suggesting that the moderate effects of DKT were observed in the early days after the operation. In a DB-RCT, Yoshikawa reported the effects of DKT after total gastrectomy for gastric cancer in elderly patients (58). DKT administration shortened the median time to the first bowel movement and resulted in fewer gastrointestinal dysfunctions on postoperative day 12. This suggests that DKT administration in the immediate postoperative period after total gastrectomy promotes early recovery of postoperative bowel function. Yaegashi et al. reported the effects of DKT on colonic motility after laparoscopic-assisted colectomy in elderly colon cancer patients (59). The DKT group had a significantly faster time until the first flatus and bowel movement and colonic transit time. This suggests DKT accelerates colonic motility in patients undergoing laparoscopic-assisted colectomy for colon cancer. Yoshikawa et al. reported the effects of DKT on the surgical inflammatory response following laparoscopic colorectal resection in an RCT that included elderly subjects (60). Postoperative DKT administration significantly suppressed the CRP level and shortened the time until first flatus. This suggests that DKT has anti-inflammatory effects and may help patients recover following surgery. Takahashi et al. reported the effects of RKT on the stasis of patients after pylorus-preserving gastrectomy in a crossover RCT that included elderly patients (61). RKT significantly reduced gastric stasis-related symptoms and improved emptying of solid meals from the remnant stomach. Endo et al. reported the effect of DKT on the stasis of patients with total gastrectomy and jejunal pouch interposition in a crossover RCT that included elderly subjects (62). DKT significantly reduced stasis-related symptoms. In the emptying test, DKT significantly accelerated emptying of both liquid and solid meals from the pouch. The pouch showed bursts of contractions, which were increased significantly by oral intake of DKT. This suggests that DKT increased intestinal motility and improved the QOL of patients with this condition. Itoh et al. reported the effects of DKT on postoperative ileus in an RCT that included elderly subjects (63). The need for further surgery was significantly reduced in patients receiving DKT. Patients receiving DKT also showed a lower tendency for recurrent ileus than those receiving the placebo. Takagi et al. reported the effects of DKT on paralytic ileus after repair of abdominal aortic aneurysm in elderly subjects (64). DKT administration significantly reduced intestinal gas. Kubo et al. reported the effects of DKT on ileus in an RCT that included elderly subjects (65). The duration to defecation, exhaust gas, and ileus tube removal did not differ significantly between the DKT and control groups. However, DKT administration reduced abdominal boating, nausea, and vomiting.

**Table 3C:** RCT of Kampo medicine for the perioperative period.

**References**	**Study design**	**Subjects (*n*)**	**Age, years (mean ± SD or range)**	**Disease/symptom**	**Kampo formulation**	**Comparator**	**Outcome**	**Adverse event related with Kampo**
Kaido et al. (66)	DB-RCT	104	56 (22–69)/57 (30–67)	Patients undergoing liver transplantation	DKT	Placebo	Postoperative total oral/enteral caloric intake was significantly accelerated in the DKT group. Portal venous flow volume and velocity were significantly higher in the DKT.	None
Nishino et al. (53)	RCT	39	68.0 (61.0–74.0)/60.5 (55.0–67.0)	Patients planned of subtotal esophageal resection for esophageal cancer	DKT	Non-DKT	The rate of body weight decreased at postoperative day 21 was significantly suppressed in the DKT group. Postoperative bowel symptoms tended to be rare in the DKT group. The serum CRP level at postoperative day 3 showing a tendency of a suppressed serum CRP level in the DKT group.	N/A
Katsuno et al. (54)	DB-RCT	71	67.7 (39–88)/68.2 (51–85)	Patients who were scheduled to undergo open surgery for sigmoid or rectosigmoid cancer	DKT	Placebo	The number of radiopaque markers in the anal side of the small intestine at 6 h was significantly greater in the DKT group.	None
Okada et al. (55)	DB-RCT	207	68.9 ± 8.4/64.9 ± 11.3	Patients who were scheduled to undergo pancreaticoduodenectomy for periampullary tumors and tumors of the head of the pancreas	DKT	Placebo	Perioperative treatment with DKT neither decreased the incidence of clinically relevant postoperative paralytic ileus nor shortened the time to first postoperative flatus.	N/A
Shimada et al. (67)	RCT	209	68(36–87)/69(31–84)	Primary and metastatic liver cancer patients who underwent hepatic resection	DKT	Placebo	DKT improve gastrointestinal dysmotility and reduce serum CRP levels in patients with grade B liver damage after hepatectomy.	None
Akamaru et al. (56)	RCT	81	63.4 ± 8.9 (32–77)/63.7 ± 9.2 (40–78)	Patients with gastric cancer scheduled for a total gastrectomy	DKT	Non-DKT	DKT significantly improved the number of stools per day, stool consistencies, and gas volume scores.	None
Katsuno et al. (57)	DB-RCT	336	68 (28–88)/69 (35–91)	Patients scheduled to undergo colectomy for colon cancer	DKT	Placebo	The frequency of bowel movement in the DKT group at postoperative day 8 was significantly lower than that in the placebo group. The moderate effects of DKT were observed early days after the operation.	None
Yoshikawa et al. (58)	DB-RCT	195	68 (33–83)/67 (28–84)	Gastric cancer patients who underwent total gastrectomy	DKT	Placebo	DKT shorter median time to first bowel movement and made fewer gastrointestinal dysfunction on postoperative day 12.	None
Yaegashi et al. (59)	RCT	51	69 (51–83)/68 (43–89)	Colon cancer patients who underwent colectomy	DKT	*Lactobacillus* preparation	DKT group had significantly faster time until first flatus and bowel movement and colonic transit time. DKT accelerated colonic motility in patients undergoing laparoscopy-assisted colectomy for colon cancer.	None
Nishi M et al. (68)	RCT	32	68.8 ± 8.7/64.3 ± 7.3	Patients who underwent hepatic resection	DKT	No DKT	DKT significantly decreased the levels of c-reactive protein and beta-(1–3)-d-glucan on postoperative day 3. DKT significantly shortened postoperative periods for the first flatus, bowel movement, and full recovery of oral intake.	None
Yoshikawa et al. (60)	RCT	30	62 ± 12 (41–80)/70 ± 5 (61–86)	Patients who underwent laparoscopic colectomy for colorectal carcinoma	DKT	Non-DKT	Postoperative DKT administration significantly suppressed CRP level and shortened the time until first flatus.	N/A
Takahashi et al. (61)	Crossover RCT	11	60 (46–70)	Pylorus-preserving gastrectomy for early gastric cancer	Rikkunshito	No rikkunshito administration	Rikkunshito significantly reduced gastric stasis-related symptoms and improved emptying of solid meals from the remnant stomach.	N/A
Endo et al. (62)	Crossover RCT	17	62 ± 10	Patients who underwent total gastrectomy with jejunal pouch interposition for gastric cancer	DKT	Non-DKT	DKT significantly reduced stasis-related symptoms. In the emptying test, DKT significantly accelerated emptying of both liquid and solid meals from the pouch. The pouch showed bursts of contractions, which were increased significantly by oral intake of DKT.	N/A
Kaiho et al. (69)	RCT	43	61.6 ± 8.1/62.4 ± 19.3/63.8 ± 10.0	Patients with liver resection	DKT	Lactulose or no administration of DKT/lactulose	DKT significantly lower postoperative serum ammonia levels with low occurrence of diarrhea.	N/A
Itoh et al. (63)	RCT	24	58 ± 10/60 ± 11	Postoperative ileus after abdominal surgery	DKT	Placebo	The need for further surgery was significantly lower in patients receiving DKT. DKT also showed a lower tendency in recurrent ileus than those receiving placebo.	N/A
Takagi et al. (64)	RCT	21	72 ± 5	Patients who underwent an aortic replacement for intrarenal abdominal aortic aneurysm with transperitoneal approach	DKT from nasogastric tube	Panthenol and/or lukewarm water from nasogastric tube	DKT significantly improved the timing and disappearance of intestinal gas.	No
Kubo et al. (65)	RCT	30	56.1 ± 22.6/53.3 ± 21.5	Simple adhesive ileus	DKT from ileus tube	Lukewarm water from ileus tube	The duration to defecation, exhaust gas, and ileus tube removal were not significantly different between DKT administration and control. However, DKT reduced abdominal bloating, nausea, and vomiting.	N/A

*DKT, daikenchuto; RCT, randomized controlled trial; N/A, not assigned*.

Kaido et al. reported the effect of DKT on oral and enteral caloric intake after liver transplantation in a DB-RCT that included elderly subjects (66). Postoperative total oral/enteral caloric intake was significantly accelerated in the DKT group. Portal venous flow volume and velocity were significantly higher in the DKT group. This suggests that postoperative administration of DKT may enhance total oral/enteral caloric intake and portal venous flow volume and velocity after liver transplantation and favorably contribute to the performance of the Enhanced Recovery After Surgery protocol. Shimada et al. reported the effect of DKT administered after hepatic resection in elderly patients with liver cancer (67). DKT improved gastrointestinal dysmotility and reduced serum CRP levels in patients with grade B liver damage after hepatectomy. This suggests that DKT is an effective and safe treatment option after hepatic resection in patients with liver cancer. Masaki et al. also reported the effect of DKT in patients who underwent hepatic resection. DKT significantly decreased the levels of CRP and beta-(1–3)-d-glucan on postoperative day 3. DKT significantly shortened postoperative periods for the first flatus, bowel movement, and full recovery of oral intake (68). Takahashi et al. reported the effect of DKT in patients with liver resection. DKT significantly lowered postoperative serum ammonia levels with low occurrence of diarrhea (69).

#### Other Diseases and Conditions Related to the Digestive System

Table 3D shows an RCT of Kampo medicine for other conditions and symptoms related with the digestive system. Bessho et al. reported the effectiveness of saibokuto (SBT) for patients with glossodynia in an RCT that included elderly subjects (70). When compared with diazepam with vitamin B complex, SBT significantly reduced the symptoms of pain, burning sensation, and discomfort.

**Table 3D:** RCT of Kampo medicine for other conditions and symptoms related with the digestive system.

**References**	**Study design**	**Subjects (*n*)**	**Age, years (mean ± SD or range)**	**Disease/symptom**	**Kampo formulation**	**Comparator**	**Outcome**	**Adverse event related with Kampo**
Bessho et al. (70)	RCT	200	61.3 (28–85)	Glossodynia	Saibokuto	Diazepam with vitamin B complex	Symptom of pain, burning sensation, and discomfort were significantly reduced compared with control.	N/A
Okabayashi et al. (71)	RCT	24	68.3 ± 7.5/68.1 ± 7.6	Patients with obstructive jaundice who received percutaneous transhepatic cholangio-drainage	Inchinkoto with drainage	Drainage	Inchinkoto significantly improved jaundice following biliary drainage and also improved subjective symptoms such as loss of appetite and general fatigue.	N/A
Miyazaki et al. (72)	RCT	16	52.3 (31–72)	Dry mouth patients who received oxybutynin hydrochloride	Ninjinyoeito added to oxybutynin hydrochloride	Oxybutynin hydrochloride	Ninjinyoeito addition reduced the symptoms of dry mouth. Gum test also showed improvement of saliva symptoms with ninjinyoeito administration.	N/A

*RCT, randomized controlled trial; N/A, not assigned*.

In an RCT, Okabayashi et al. reported the effects of inchinkoto (ICKT) on the bilirubin reduction rate after biliary drainage in elderly patients with obstructive jaundice (71). ICKT significantly improved jaundice following biliary drainage and also improved subjective symptoms such as loss of appetite and general fatigue. Miyazaki et al. reported the efficacy of ninjinyoeito (NYT) for dry mouth induced by oxybutynin hydrochloride to treat psychogenic frequency or unstable bladder in an RCT that included elderly subjects (72). The addition of NYT reduced the symptom of dry mouth in 75% of patients. Saliva secretion also improved after NYT addition.

### Kampo Medicine for Symptoms of Dementia

Dementia has become a global health issue. The number of people living with dementia in the world was estimated to be 46.8 million in 2015 (73). Japan has one of the most rapidly aging societies; the prevalence of dementia was already beyond 3% (five million) in 2015, and it has been increasing, even though the Japanese population has begun to decline (74). This situation might drive doctors to conduct a number of clinical studies using Kampo medicine for the symptoms of dementia. Dementia is a syndrome associated with declines in memory, thinking, behavior, and the ability to perform daily activities. Cognitive disorders and noncognitive symptoms, that is, behavioral and psychological symptoms of dementia (BPSD), are equally important clinical manifestations. Kampo medications are composed of multiple herbal ingredients and have different target symptoms. Therefore, based on the current clinical evidence, we herein introduce some Kampo medications and their target symptoms (Table 4).

**Table 4:** RCT of Kampo medicine for symptoms of dementia.

**References**	**Study design**	**Subjects (n)**	**Age, years (mean ± SD)**	**Disease/symptom**	**Kampo formulation**	**Comparator**	**Outcome**	**Adverse event related with Kampo**
Shimada et al. (75)	RCT	57	78.9 ± 7.6	VD and subarachnoid hemorrhage	CTS	Placebo	The scores of cognitive dysfunction (Hasegawa's Dementia Scale-Revised), the global improvement rating, utility rating, global rating for subjective symptoms, subjective symptoms (shoulder stiffness and palpations), global rating for psychiatric symptoms, psychiatric symptoms (decline in interest in television or books, lack of facial expression), and ADL were decreased after 12-week administration of CTS compared to placebo.	1 liver dysfunction in 1 case
Terasawa et al. (76)	DB-RCT	139	76.6 ± 8.4	VD	CTS	Placebo	The global improvement rating, global rating for subjective symptoms, psychiatric symptoms (decline in simple arithmetic ability, global intellectual ability, sleep disturbance, hallucination, and delusion), and ADL were alleviated after 12-week administration of CTS compared to placebo.	Urtic aria in one, diarrhea in one, appetite, in one loss, oral bitterness in one, and liver dysfunction in one case (no information of intervention group)
Suzuki et al. (78)	DB-RCT	30	84.4 ± 6.3	AD and VD	CTS/GJG	Placebo	CTS improved the scores of cognitive dysfunction (MMSE), and ADL, compared to placebo.	N/A
Iwasaki et al. (79)	DB-RCT	33	84.4 ± 7.8	Mixed dementia and AD	HJG	Placebo	Cognitive dysfunction (MMSE), and ADL were improved after treatment of HJG compared to placebo.	No
Maruyama et al. (77)	Observer blind RCT	38	73.7 ± 5.6 74.6 ± 3.9	AD	Kamiuntanto add to donepezil	Donepezil	Kamiuntanto added to donepezil improved the scores of MMSE, and Alzheimer's Disease Assessment Scale, compared to donepezil alone.	No
Higashi et al. (81)	RCT	75	82.8 ± 8.1 84.2 ± 6.4 86.1 ± 5.0	AD	KHT/GJG	No treatment	The scores of MMSE improved only in the KHT treatment group. The orientation and attention subscale scores of the MMSE improved significantly in the KHT-treatment group.	N/A
Iwasaki et al. (82)	Observer blind RCT	52	80.3 ± 9.0	AD, VD, mixed dementia and DLB	YKS	No treatment	Four-week administration of YKS significantly improved BPSD, especially in hallucinations, agitation/aggression, irritability/lability, and aberrant motor activity, and ADL.	No
Mizukami et al. (83)	RCT- cross over	*N* = 88	78.7 ± 5.4	AD and DLB	YKS	No treatment	BPSD, especially in agitation/aggression and irritability/lability subscale scores, were improved after treatment of YKS.	Gastrointestinal distress in 3 cases
Monji et al. (84)	RCT	*N* = 15	80.2 ± 4.0	AD	YKS add to sulpiride	Sulpiride	The average dose of sulpiride tened to be less in the YKS treatment group than the control group.	Hypokalemia in 2 cases
Okahara et al. (85)	RCT	*N* = 61	76.1 ± 8.1 77.1 ± 6.8	AD	YKS add to donepezil	Donepezil	The scores of BPSD decreased in the YKS treatment group. The subscale score of agitation and irritability decreased significantly.	N/A
Teranishi et al. (86)	Rater blind RCT	*N* = 76	83.5 ± 5.8 80.7 ± 8.8 83.2 ± 5.4	AD, VD, and DLB	YKS	Risperidone/fluvoxamine	The three intervention significantly alleviated BPSD. The adverse events were more frequent in the risperidone-treatment group.	No
Fukuhara et al. (87)	DB-RCT	*N* = 145	78.3 ± 5.4 78.5 ± 5.1	AD	YKS	Placebo	The BPSD scores did not change in both YKS and placebo intervention. The subgroup scoring below 20 points on the MMSE at baseline showed a greater improvement in BPSD, especially in agitation/aggregation in the YKS-treatment group, compared to the placebo group. In the subgroup younger than 74 years of age, a significant decrease in the score for agitation/aggression was shown in the YKS-treatment group.	Hypokalemia in 3 cases

*VD, Vascular dementia; CTS, chotosan; ADL, activity of daily living; AD, Alzheimer's disease; GJG, goshajinkigan; HJG hachimijiogan; MMSE, Mini-Mental State Examination; KHT, kihito; DLB, dementia with Lewy body; YKS, yokukansan; BPSD, behavioral and psychological symptoms of dementia; N/A, not assigned; SD, standard deviaton*.

#### Cognitive Disorders

Chotosan (CTS) was originally used for headache, tinnitus, and dizziness. In 1994, Shimada et al. conducted a multicenter placebo-controlled RCT using CTS (75). After 12 weeks of CTS treatment, patients with vascular dementia had a decrease in the score of cognitive dysfunctions (Hasegawa's Dementia Scale-Revised) when compared to baseline. CTS was superior to the placebo in the global improvement rating, utility rating, global rating for subjective symptoms, subjective symptoms (shoulder stiffness and palpations), global rating for psychiatric symptoms, psychiatric symptoms (decline in interest in television or books and lack of facial expression), and activities of daily living (ADLs). Terasawa et al. conducted another placebo-controlled RCT using CTS for vascular dementia (76). When compared with the placebo, the global improvement rating, global rating for subjective symptoms, psychiatric symptoms (decline in simple arithmetic ability, global intellectual ability, sleep disturbance, hallucination, and delusion), and ADLs significantly improved after a 12-week administration of CTS. In the study, cognitive dysfunctions did not improve. The overall safety rating did not differ significantly between the chitosan treatment group and the placebo group. Suzuki et al. reported that an 8-week treatment of CTS improved cognitive dysfunction [assessed by the Mini-Mental State Examination (MMSE)] and ADL when compared to the baseline in patients with Alzheimer's disease; goshajinkigan (GJG) treatment and the placebo did not improve these symptoms (77). In 2017, Imai et al. conducted a meta-analysis of the three above-mentioned RCTs to assess the effectiveness and acceptability of CTS (78). CTS was more effective than the placebo for short-term improvement of cognitive function. The acceptability, measured in terms of the number of dropouts due to adverse effects, did not differ between the CTS treatment group and the placebo group. However, the results are considered imprecise, partly because of the small number of participants. Iwasaki et al. conducted a placebo-controlled DB-RCT using hachimijiogan (HJG), a pill made with herbs and honey, for the treatment of dementia (79). Administration of HJG for 8 weeks significantly improved cognitive dysfunction (assessed by MMSE) and ADL (assessed by the Barthel index) when compared to baseline, while the placebo did not change those scores. No adverse events were observed. Maruyama et al. reported the effectiveness of a combination of donepezil and kamiuntanto on cognitive function and brain perfusion in patients with Alzheimer's disease (80). A 12-week observer-blinded RCT revealed that combination treatment with a donepezil and kamiuntanto decoction significantly improved cognitive function (MMSE and ADAS-cog) when compared with treatment with donepezil alone. Furthermore, cerebral blood flow in the frontal region (measured by single photon emission computed tomography) significantly increased in the combination treatment group. In 2007, Higashi et al. reported the effectiveness of kihito (KHT) extract granules on the cognitive function of patients with Alzheimer's disease (81). The MMSE showed significant improvement 3 months after treatment with KHT, but not in the nontreated or GJG-treated groups. The orientation and attention subscale scores of the MMSE improved significantly in the KHT treatment group when compared with those of the nontreatment group. No adverse events were observed in any of the groups.

#### BPSD

Yokukansan (YKS) was originally used in children for the treatment of agitation and crying at night. Starting in the 1980s, when the Japanese society shifted to an aging society, YKS began to be used for the treatment of BPSD. Five RCTs and one meta-analysis have shown the efficacy of YKS for BPSD, especially for delusions, hallucinations, and agitation/aggression. In 2005, Iwasaki et al. firstly conducted a multicenter RCT using YKS for dementia patients (82). A 4-week administration of YKS significantly improved BPSD [assessed by the Neuropsychiatric Inventory (NPI)], especially hallucinations, agitation/aggression, irritability/lability, and aberrant motor activity. YKS also improved ADL (assessed by the Barthel index). In an RCT conducted by Mizukami et al., 88 dementia patients received 4 weeks of YKS treatment and spent another 4 weeks under observation (no treatment) in a crossover design (83). BPSD improved in the YKS treatment period, and no rebound phenomenon was observed in the following observation period. Monji et al. reported that 12 weeks of YKS treatment significantly improved BPSD in patients with Alzheimer's disease (84). The average dose of antipsychotics (sulpiride) tended to be less in the YKS treatment group than in the control group. The Barthel index did not change in the YKS treatment group or the control group. In 2010, Okahara et al. reported the efficacy of 4 weeks of treatment with YKS and donepezil for BPSD in patients with Alzheimer's disease (85). Among the NPI subscales, the agitation and irritability scores decreased significantly. Cognitive dysfunction, ADL, and caregiver burden scores did not change in the YKS treatment group or in the control group. Teranishi et al. reported the efficacy and safety of YKS compared with risperidone and fluvoxamine for BPSD in patients with dementia (86). All three drugs significantly alleviated BPSD, with no significant intergroup differences. The tolerability analysis revealed that adverse effects (constipation, muscle rigidity, and extrapyramidal symptoms) were more frequent in the risperidone treatment group. In 2016, Furukawa et al. conducted a placebo-controlled DB-RCT on patients with Alzheimer's disease (87). Both 4 weeks of YKS treatment and the placebo improved BPSD, with no significant intergroup differences. The subgroup scoring below 20 points on the MMSE at baseline showed a greater improvement in BPSD, especially in agitation/aggregation in the YKS treatment group, when compared to the placebo group. In the subgroup younger than 74 years of age, a significant decrease in the subcategory score for agitation/aggression was shown in the YKS treatment group when compared with the placebo group. In 2016, Matsunaga et al. conducted a meta-analysis of the above-mentioned RCTs using YKS for BPSD in dementia patients (88). YKS significantly decreased total BPSD scores when compared with the controls (placebo or usual care), especially the subscale scores for delusions, hallucinations, and agitation/aggression. However, only in the Alzheimer's disease patients, YKS was not superior to the controls for BPSD. YKS treatment significantly improved ADL when compared with the controls. MMSE scores did not improve in the YKS treatment group or in the control group. Incidence of adverse effects did not differ significantly between the YKS treatment and control groups. Various Kampo formulations are clinically effective for the treatment of dementia. A Kampo medicine may be selected according to the patients' symptoms. Adverse events due to Kampo medicine are not frequent. Therefore, Kampo medicine may be a treatment option for both cognitive dysfunction and BPSD.

### Kampo Medicine for Pain Control

In elderly individuals, physical, psychological, and social changes cause various types of chronic pain. Western analgesic medications are used as the basic approach for pain relief; however, modulation of organ systems and pharmacokinetics often induce adverse effects in aging patients. Furthermore, chronic pain often accompanies various symptoms such as coldness, fatigue, and depression. These conditions exacerbate pain and hinder the physical exercise needed to control pain.

Kampo medicine balances the equilibrium of mind and body disturbed due to external and internal factors. As a result, it is possible to relieve pain as well as multiple coexisting symptoms. In Japan, Kampo medicine is empirically assumed to be effective and widely applied for the treatment of pain. However, the suitable formula often differs depending on the patient's personality. This inhibits the performance of large clinical trials; most studies are case reports or case series (Table 5). However, animal studies have recently begun to elucidate the mechanisms of Kampo formulae.

**Table 5:** RCT of Kampo medicine for pain.

**References**	**Study design**	**Subjects (*n*)**	**Age, years (mean ± SD)**	**Disease/symptom**	**Kampo formulation**	**Comparator**	**Outcome**	**Adverse reaction**
Nakae et al. (101)	RCT	162	60(16–90) 66(23–90)	Rib fracture	JDI	NSAIDs	Shorten the duration, Lower healthcare expenditure	No adverse reaction in JDI, gastrointestinal symptoms in 2.5% of the NSAIDs group
Majima et al. (104)	RCT	47	68.3 ± 10.0 71.5 ± 6.0	Osterarthritis of knee	BOT	Loxoprofen	Improve the Knee Society Rating System and SF-36	Dry month in a patient
Watanabe et al. (114)	RCT	116	59.4 ± 7.8 60.9 ± 7.4	Diabetes mellitus type 2	GJG	No GJG	Decrease progression neuropathy	None

*JDI, jidabokuippo; BOT, boiogito; GJG, goshajinkigan; NSAIDs, non-steroidal anti-inflammatory drugs; SD, standard deviaton*.

#### Musculoskeletal Pain

##### Back Pain

Back pain has a prevalence of 24.4% in the Japanese population over 70 years of age (89). Degenerative spine conditions (spondylosis, spinal stenosis, interval disc disease, etc.) and osteoporosis (OP) induce skeletal deformities, joint imbalance, and tension in muscular structures (90), which lead to chronic musculoskeletal pain.

GJG is used to alleviate symptoms in the lower part of the body associated with aging. Hamaguchi et al. reported the efficacy of routine GJG administration for low back pain (LBP) (91). In a retrospective observational study, LBP improved within 6 months in 10 out of 28 patients. Patients with spinal stenosis were less likely to respond to GJG than those without spinal disease. GJG is expected to relieve LBP in patients without spinal disease. In a retrospective cohort study, Oohata et al. reported the efficacy of Kampo medicine in patients with lumbar spinal stenosis (92). Patients received routine medication with or without Kampo treatment. The frequently used Kampo medicines were GJG, HJG, and shakuyakukanzoto. The rate of reduced and discontinued use of pregabalin and opioid was significantly greater in the Kampo treatment group than in the non-Kampo treatment group. Side effects were observed in 6.3% of patients treated with Kampo medicine and in 62.5% of patients treated without Kampo medicine. Hamaguchi et al. reported the efficacy of Kampo medicine for symptoms in the lower extremities caused by various lumbar spinal diseases (93). In a retrospective observational study, the addition of Kampo medicine to Western medications relieved pain in 53% of patients and relieved coldness in 50% of patients. On the other hand, numbness was improved in only 21% of patients. The effective formulae included shimbuto, keishikajutsubuto (KSTJB), ryokeijutsukanto, and GJG. Coldness is an uncomfortable symptom that exaggerates chronic pain. Takahashi et al. reported the effectiveness and safety of tokishigyakukagoshuyushokyoto (TSGS) for improving coldness with LBP (94). This retrospective observation study showed that 74% of patients were satisfied with the relief from coldness. The VAS significantly decreased from 57.7 ± 11.4 to 43.7 ± 14.1. Routine treatment combined with Kampo medicine may be safer and more effective than treatment using only Western medicines. There are some case reports on back pain. In four case reports, Western treatment involving nerve block could not relieve the pain of geriatric patients with lumbar spinal stenosis. However, Kampo treatment was successful (95–98). In these case reports, some patients were able to avoid surgery. Patients often suffer from residual symptoms after operations for spinal diseases. Ogawa et al. reported a case of postoperative residual pain, coldness, and numbness treated with Kampo medicine (99).

OP-induced fractures result in severe pain. Tetsumura et al. reported a case of multiple OP-induced fractures treated with Kampo medicine (100). KSTJB and other Kampo formulae diminished the suffering of a bedridden patient. The patient was able to stand 11 months after treatment. Nakae et al. performed an RCT to compare the efficacies of jidabokuippo (JDI) and nonsteroidal anti-inflammatory drugs (NSAIDs) in young and old patients with rib fracture (101). The treatment duration was significantly shorter in the JDI group than in the NSAID group. Furthermore, healthcare expenditure was significantly lower in the JDI group than in the NSAID group.

#### Osteoarthritis

Osteoarthritis (OA) has a prevalence of 32.5% in the Japanese population over 60 years of age (102). The pain of OA is attributed to unstable joint structure, anatomical degeneration, and inflammation (103). Though standard pharmacotherapy is used for nociceptive pain, it is sometimes ineffective. Boiohito (BOT) is often used for arthritis of the knee. In an RCT, Majima et al. reported the clinical efficacy of BOT on OA of the knee (104). Patients were assigned to the concomitant-use group (both loxoprofen and BOT) and the loxoprofen group (loxoprofen alone). The knee score, based on the Knee Society Rating System and the 36-item short form from the Medical Outcome Study Questionnaire (SF-36), improved in both groups. However, the score for the ability to climb up and down a staircase, based on the Knee Society Rating System functional score and joint fluid, was significantly improved in the concomitant-use group compared to the loxoprofen group.

Bushi is a crude drug with an analgesic effect. In a nonrandomized prospective study, Nakae reported the efficacy and safety of bushimatu (powdered processed aconite root) for the treatment of pain associated with orthopedic disease (105). OA of the knee was the most common orthopedic disease. Patients were administered bushimatu (1.5–8 g/day) with other Kampo formulae without NSAIDs. Patients with ≥50% and ≥25% reductions in VAS accounted 102 and 84 out of 257 patients, respectively, 4 weeks after treatment. Three patients (1.2%) experienced side effects.

#### Neuropathic Pain

Neuropathic pain develops after difficult-to-treat injury of neurons along nociceptive pathways. YKS has a variety of neuropharmacological actions, such as neuroprotection, anti-stress effect, promotion of neuroplasticity, and anti-inflammatory effect (106). Therefore, YKS is sometimes used to treat neuropathic pain. Nakamura et al. reported 11 cases (36–85 years old) of successful treatment of neuropathic pain [postherpetic neuralgia (PHN), central pain, complex regional pain syndrome, and trigeminal neuralgia] using YKS (107). The patients had VAS scores of 17–81 despite Western conventional treatment. The VAS scores decreased to 0–22 after YKS administration for 2 days to 2 months.

PHN is a persisting neuropathic pain syndrome that occurs after resolution of a herpes zoster (HZ) rash. The frequency increases with age, occurring in 20% of people aged 60–65 years and in more than 30% of people aged >80 years who had acute HZ (108). Nakabayashi et al. published a case series on medication combined with KBG and bushimatsu for patients with PHN (2–92 months after HZ onset) (109). The VAS score improvement rate was 76.5 ± 27.7%. However, three of 15 patients could not continue the study due to hot flashes and gastric discomfort. There are some reports of successful PHN treatment with Kampo medicine (110–112). In these reports, patients had suffered from PHN from 2 months to 2 years. From 8 weeks to 4 months after treatment, their symptoms disappeared with Kampo medicine. However, in some cases, pain worsened again when the Kampo medication was discontinued. Radical treatment of PHN may be difficult, but it may be effective if Kampo medication is started during the acute stage of herpes infection.

In elderly people, the prevalence of diabetes increases due to glucose intolerance. Diabetic neuropathy is the most common chronic complication, with an estimated lifetime prevalence exceeding 50% (113). In an RCT, Watanabe et al. reported the efficacy of GJG on the progression of type 2 diabetes complications in middle-aged and older people (114). GJG significantly decreased glycated hemoglobin and progression of neuropathy (ankle reflex) when compared with the control. GJG is also used to prevent and relieve peripheral neuropathy due to chemotherapy (115).

### Kampo Medicine for Others

#### Peripheral Arterial Disease

Peripheral arterial disease (PAD) represents atherosclerotic disease associated with aging. PAD has a prevalence of 15–20% in the Japanese population over 70 years of age (116). The clinical presentation of a reduction in limb blood flow includes peripheral coldness, atypical leg pain, or intermittent claudication; as it progresses, it may present with ischemic ulcer or critical limb ischemia.

In a prospective study, Kawago et al. reported the efficacy of HJG for improvement of the QOL in patients with PAD (117). The patients were administered HJG for 6 months without any new interventions. The pain score on the Japanese version of the Walking Impairment Questionnaire (WIQ) improved from 25.0 (0.0–50.0) at baseline to 75.0 (68.8–100.0). The absolute change was 37.5 (25.0–75.0). TSGS improved peripheral blood flow and perception of peripheral coldness (118). In a nonrandomized prospective study, Jojima reported the efficacy of TSGS for arteriosclerosis obliterans (ASO) (119). TSGS and cilostazol improved the absolute claudication distance 1 and 3 months after treatment. However, side effects were observed in 4% of patients treated with TSGS, while they were observed in 38% of patients treated without Kampo. One case report has been published regarding Kampo treatment for severe limb pain with ASO (120). A decoction of KBG and daisaikoto relieved pain, coldness, and ischemic ulcers and eliminated the need for limb amputation.

#### Rehabilitation

Physical exercise is necessary to improve pain and prevent secondary injuries. However, elderly individuals often cannot take exercise sufficiently due to frailty or sarcopenia.

Hozai is one group of Kampo formulations that restore vitality to patients who have lost psychological and physical energy due to various diseases or aging (121). These formulations improve pain in various conditions induced by sarcopenia and frailty, such as fatigue, anorexia, and mental problems. Sakamoto et al. reported their experience of using Kampo, mainly Hozai formulae (RKT, HET, NYT, etc.) for rehabilitation (122). In a prospective non-RCT, Sakisake et al. reported the efficacy of NYT against frailty (123). Administration of NYT for 24 weeks prevented deterioration of muscle mass and muscle quality score when compared to the control group. Furthermore, the NYT group significantly improved grip strength, whereas there was no change in the control group.

## Discussion

**Figure 1:**
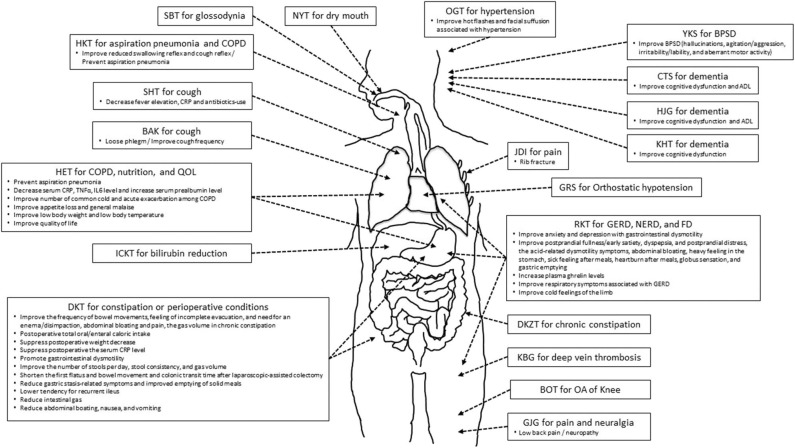
The relationship between Kampo medicines and symptome/condition/disorder in organs and viscera.

Here, we reviewed RCTs on the efficacy of Kampo medicine for GS. Figure 1 shows the relationship between Kampo medicines and organs and physiological systems. One of the characteristics of Kampo medicine is the use of multiple crude drugs (Table 6). Therefore, Kampo medicine can act upon multiple organs and physiological systems. HET is effective for COPD, nutrition, anti-inflammation, and QOL; RKT for GERD, NERD, FD, and appetite; and DKT for constipation and perioperative conditions. As GS symptoms are expressed by the 3Ms or 4Is, Kampo medicine can contribute to GS.

**Table 6:** Kampo medicines and their crude drugs by RCT.

**Kampo medicine**	**Indication as per the Japanese Pharmacopeia (i.e.,)**	**Included crude drugs**											
Orengedokuto (OGT)	Patients with ruddy face with comparatively strong constitution, a touch of hot flushes, and a tendency to irritability: nose bleeding, hypertension, insomnia, neurosis, gastritis, alcoholic hangover, climacteric disturbance and automatic imbalance syndrome peculiar to women resembling climacteric disturbance, dizziness, palpitation, eczema or dermatitis, and pruritus cutaneous	JP Scutellaria Root	JP Coptis Rhizome	JP Gardenia Fruit	JP Phellodendron Bark								
Goreisan (GRS)	Patients with oral dryness and decreased urine volume: edema, nephrosis, alcoholic hangover, acute gastrointestinal catarrh, diarrhea, nausea, vomiting, dizziness, water retention in the stomach, headache, uremia, heat-stroke, and diabetes mellitus	JP Alisma Rhizome	JP Atractylodes Lancea Rhizome	JP Polyporus Sclerotium	JP Poria Sclerotium	JP Cinnamon Bark							
Keishibukuryogan (KBG)	Patients with solid constitution who have ruddy face and generally solid abdomen with resistance in the lower abdomen: inflammation in the uterus and its adnexa, endometritis, menstrual irregularity, dysmenorrhea, leukorrhea, climacteric disturbance (headache, dizziness, feeling of hot flushes, shoulder stiffness, etc.), oversensitivity to cold, peritonitis, contusion, hemorrhoid, and orchitis	JP Cinnamon Bark	JP Peony Root	JP Peach Kernel	JP Poria Sclerotium	JP Moutan Bark							
Rikkunshito (RKT)	Patients with weak stomach, loss of appetite and full stomach pit, and those who are easily fatigued, anemic and likely to have cold limbs: gastritis, gastric atony, gastroptosis, maldigestion, anorexia, gastric pain, and vomiting	JP Atractylodes Lancea Rhizome	JP Ginseng	JP Pinellia Tuber	JP Poria Sclerotium	JP Jujube	JP Citrus Unshiu Peel	JP Glycyrrhiza	JP Ginger				
Daikenchuto (DKT)	Abdominal cold feeling and pain accompanied by abdominal flatulence	JP Processed Ginger	JP Ginseng	JP Zanthoxylum Fruit	JP Koi								
Daiokanzoto (DKZT)	Constipation	JP Rhubarb	JP Glycyrrhiza										
Saibokuto (SBT)	Patients who have depressed feelings and a feeling of foreign body in the throat and esophagus and who sometimes have palpitation, dizziness, nausea, etc.: infantile asthma, bronchial asthma, bronchitis, coughing, and anxiety neurosis	JP Bupleurum Root	JP Pinellia Tuber	JP Poria Sclerotium	JP Scutellaria Root	JP Magnolia Bark	JP Jujube	JP Ginseng	JP Glycyrrhiza	JP Perilla Herb	JP Ginger		
Ninjinyoeito (NYT)	Declined constitution after recovery from disease, fatigue and malaise, anorexia, perspiration during sleep, cold limbs, and anemia	JP Rehmannia Root	JP Japanese Angelica Root	JP Atractylodes Rhizome	JP Poria Sclerotium	JP Ginseng	JP Cinnamon Bark	JP Polygala Root	JP Peony Root	JP Citrus Unshiu Peel	JP Astragalus Root	JP Glycyrrhiza	JP Schisandra Fruit
Inchinkoto (ICKT)	Patients with a comparatively strong constitution and decreased urine volume who are somewhat likely to have constipation: jaundice, hepatic cirrhosis, nephrosis, urticaria, and stomatitis	JP Artemisia Capillaris Flower	JP Gardenia Fruit	JP Rhubarb									
Chotosan (CTS)	Chronic headache with hypertension in those middle-aged or elderly	JP Gypsum	JP Uncaria Hook	JP Citrus Unshiu Peel	JP Ophiopogon Tuber	JP Pinellia Tuber	JP Poria Sclerotium	JP Chrysanthemum Flower	JP Ginseng	JP Saposhnikovia Root	JP Glycyrrhiza	JP Ginger	
Hachimijiogan (HJG)	Patients with severe fatigue or malaise, decreased urinary output or increased urinary frequency, dry mouth, and alternate cold and hot feeling in the extremities: nephritis, diabetes mellitus, impotence, sciatica, low back pain, beriberi, cystorrhea, prostatic hypertrophy, and hypertension	JP Rehmannia Root	JP Cornus Fruit	JP Dioscorea Rhizome	JP Alisma Rhizome	JP Poria Sclerotium	P Moutan Bark	JP Cinnamon Bark	JP Powdered Processed Aconite Root				
Kihito (KHT)	patients with a delicate constitution and a poor complexion: anemia and insomnia	JP Astragalus Root	JP Jujube Seed	JP Ginseng	JP Atractylodes Rhizome	JP Poria Sclerotium	JP Longan Aril	JP Polygala Root	JP Jujube	JP Japanese Angelica Root	JP Glycyrrhiza	JP Ginger	JP Saussurea Root
Yokukansan (YKS)	Patients with delicate constitution and nervousness: neurosis, insomnia, night cry in children, and peevishness in children	JP Atractylodes Lancea Rhizome	JP Poria Sclerotium	JP Cnidium Rhizome	JP Uncaria Hook	JP Japanese Angelica Root	JP Bupleurum Root	JP Glycyrrhiza					
Hangekobokuto (HKT)	Patients who have depressed feelings and a feeling of foreign body in the throat and esophagus and who sometimes have palpitation, dizziness, nausea, etc.: anxiety neurosis, nervous gastritis, hyperemesis gravidarum, coughing, hoarseness, nervous esophageal stricture, and insomnia	JP Pinellia Tuber	JP Poria Sclerotium	JP Magnolia Bark	JP Perilla Herb	JP Ginger							
Bakumondoto (BAK)	Coughing with a hard, obstructive sputum, bronchitis, and bronchial asthma	JP Ophiopogon Tuber	JP Brown Rice	JP Pinellia Tuber	JP Jujube	JP Glycyrrhiza	JP Ginseng						
Goshajinkigan (GJG)	Patients with decreased urine volume or polyuria sometimes having dry mouth who are easily fatigued and easily feel cold in the extremities: leg pain, low back pain, numbness, blurred vision in old patients, pruritus, dysuria, frequent urination, and edema	JP Rehmannia Root	JP Achyranthes Root	JP Cornus Fruit	JP Dioscorea Rhizome	JP Plantago Seed	JP Alisma Tuber	JP Poria Sclerotium	JP Moutan Bark	JP Cinnamon Bark	JP Powdered Processed Aconite Root		
Jidabokuippo (JDI)	Swelling and pain caused by contusion	JP Cnidium Rhizome	JP Atractylodes Lancea Rhizome	JP Forsythia Fruit	JP Lonicera Leaf and Stem	JP Saposhnikovia Root	JP Glycyrrhiza	JP Schizonepeta Spike	JP Safflower	JP Rhubarb			
Boiogito (BOT)	Patients with a white-complexion, soft muscles, and a flabby constitution who are easily fatigued, perspire profusely, do not excrete enough urine, and develop edema in the lower limbs and swelling and pain of the knee joint: nephritis, nephrosis, nephropathy of pregnancy, hydrocele testis, obesity, arthritis, carbuncle, furuncle, myositis, edema, dermatosis, hyperhidrosis, and menstrual irregularity	JP Astragalus Root	JP Sinomenium Stem	JP Atractylodes Lancea Rhizome	JP Jujube	JP Glycyrrhiza	JP Ginger						

The possible mechanisms of Kampo medicines have been reported in recent years. For example, YKS is composed of seven crude drugs (Table 6) and has been used to improve irritation, insomnia, muscle twitching, and pain. Several studies reported various neuropharmacological actions of YKS, namely, on serotonergic, glutamatergic, cholinergic, dopaminergic, adrenergic, and gamma-aminobutyric acidergic neural systems (124). These actions maintain neural signal conduction and neuronal function of neurons as well as glial cells (125). GJG is composed of 10 crude drugs and has been used to alleviate various types of age-related conditions, including muscle weakness of the lower limbs, dysuria, foot edema, and cold sensation of the lower limbs. Recently, GJG is used to prevent and relieve various types of peripheral neuropathy. GJG has antinociceptive effects via increasing produced nitric oxide (126), reduces hypersensitivity by suppressing the overexpression of TRPM8 and TRPA1 mRNA (127), and ameliorates allodynia via the suppression of TNF-α expression in the spinal cord (128). Furthermore, GJG has also been reported to suppress sarcopenia via the insulin growth factor-1/insulin pathway, maintains the expression of mitochondrial-related transcription factors, and suppresses the expression of TNF-α (129).

DKT is composed of four crude drugs and has been used to treat abdominal pain and abdominal bloating with abdominal coldness. DKT treats abdominal symptoms by enhancing the secretions of motilin (130), substance-P, calcitonin gene-related peptide, and adrenomedullin (131–133) and activating the transient receptor potential of the vanilloid receptor complex (134). RKT is composed of eight crude drugs and has been used to treat appetite loss, upper abdominal discomfort, and indigestion. A recent study reported that RKT increases plasma ghrelin levels in humans and mice (135) and restores decreased plasma ghrelin levels induced by serotonin release in rats. HKT is composed of five crude drugs and has been used for pharyngeal discomfort. It has been reported that HKT modulates cerebral levels of 5-hydroxytryptamine, noradrenaline, and dopamine in mice (20).

The efficacy and safety of Kampo medicine were investigated in several clinical studies. Based on these reports, clinical practice guidelines have recommended Kampo medicines for symptoms in geriatrics. Our previous study (136), conducted between January 1, 2012, and October 31, 2017, showed that the Clinical Practice Guideline for the Pruritus Cutaneous Universalis (137), Practical Guideline for the Management of Allergic Rhinitis in Japan (138), the Japanese Respiratory Society Guidelines for the Management of Cough (139), Evidence-Based Clinical Practice Guidelines for GERD (140), Evidence-Based Clinical Practice Guidelines for Functional Dyspepsia (141), Evidence-Based Clinical Practice Guidelines for Irritable Bowel Syndrome (142), Evidence-Based Clinical Practice Guidelines for Chronic Constipation (143), Clinical Guidelines for Overactive Bladder Syndrome (144), and Practice Guideline for Dementia (145) have recommended the use of Kampo medicines for skin symptoms, allergy, cough, gastrointestinal dysfunction, urinary dysfunction, and dementia.

In recent years, the usefulness of Kampo medicine in the clinical setting has been investigated using the diagnosis procedure combination (DPC) inpatient database in Japan (Table 7). A propensity score analysis using DPC is a retrospective investigation; however, the groups of patients with or without intervention can be matched, and the subject number is large. Thus, this method can show the intervention's effect and influence on the social economy. Jo et al. reported a reduction in the exacerbation of COPD in patients of advanced age using DKT (33). DKT users had a significantly lower risk of rehospitalization or death after discharge. Subgroup analysis of long-acting muscarinic receptor antagonist users showed a significant difference in rehospitalization or death, while subgroup analysis of long-acting muscarinic receptor antagonist nonusers showed no significant difference. Yasunaga reported the effects of GRS on reoperation rates after burr-hole surgery for chronic subdural hematoma (146). GRS use was significantly associated with a lower reoperation rate when compared with nonuse. These results suggest that GRS use reduced the need for reoperation after burr-hole surgery for chronic subdural hematoma. Yasunaga et al. also reported effects of DKT on postoperative adhesive small bowel obstruction requiring long-tube decompression (LTD) (147). DKT use was associated with a significantly shorter duration of LTD, a shorter duration between long-tube insertion and discharge, and lower hospital charges when compared with DKT nonuse. This suggests that DKT effectively reduces the duration of LTD and saves costs.

**Table 7:** Propensity score analysis of Kampo medicine.

**References**	**Study design**	**Subjects (*n*)**	**Age, years (mean ± SD)**	**Disease/symptom**	**Kampo formulation**	**Comparator**	**Outcome**
Jo et al. (146)	Propensity score analysis	2385	82.1 ± 4.8 82.1 ± 4.8	Chronic obstructive pulmonary disease exacerbation	DKT	No DKT	DKT users had a significantly lower risk of re-hospitalization or death after discharge. Subgroup analysis of long-acting muscarinic receptor antagonists users showed a significant difference in re-hospitalization or death, while subgroup analysis of long-acting muscarinic receptor antagonists non-users showed no significant difference.
Yasunaga (147)	Propensity score analysis	7758	76.2 (10.7) 76.2 (10.7)	Chronic subdural hematoma	GRS	No GRS	GRS use was significantly associated with a lower reoperation rate compared with non-use.
Yasunaga et al. (148)	Propensity score analysis	288	68.4 ± 10.1 67.9 ± 9.1	Postoperative adhesive small bowel obstruction requiring long-tube decompression	DKT	No DKT	Patients who received DKT showed significant shorter duration of long-tube decompression (LTD), shorter duration between long-tube insertion and discharge, and lower hospital charges compared with patients without DKT. It suggested that DKT is effective for reducing the duration of LTD and saving costs.

*DKT, daikenchuto; GRS, goreisan; SD, standard deviation*.

Not only the efficacy but also adverse drug reactions (ADRs) were reported in RCTs of Kampo medicine (148). The total incidence of ADRs was 2.47%, and those of pseudoaldosteronism and liver disorders caused by Kampo medicine were 0.02 and 0.16%, respectively. In our previous study, the incidence of ADRs was 0.09% for BAK, 0.44% for DKT, 2.04% for RKT, 1.7% for GJG, 3.45% for HET, 3.34% for CTS, 4.41% for NYT, and 5.17% for YKS. Many of the ADRs were gastrointestinal disorders.

Due to an increase in Japan's “super-aging population” and a decline in the country's birth rate, medical expenses are expected to increase and pose an important problem. Furthermore, medical expenses have grown every year. This review has shown the efficacy, safety, and the social and economic advantages associated with Kampo treatment.

## Author Contributions

ST designed this report. ST, NT, RO, RA, and AK collected and selected the articles and wrote the manuscript. TI revised the manuscript.

## Conflict of Interest

ST, AK, and TI belong to the Department of Kampo and Integrative Medicine at Tohoku University School of Medicine. The department received a grant from Tsumura, a Japanese manufacturer of Kampo medicine; however, the grant was used as per Tohoku University rules. Potential conflicts of interests were addressed by the Tohoku University Benefit Reciprocity Committee and were managed appropriately. The remaining authors declare that the research was conducted in the absence of any commercial or financial relationships that could be construed as a potential conflict of interest.

## Abbreviations

BAK: Bakumondoto
BOT: Boiogito
CTS: Chotosan
DKT: Daikenchuto
DKZT: Daiokanzoto
GRS: Goreisan
GJG: Goshajinkigan
HJG: Hachimijiogan
HKT: Hangekobokuto
HET: Hochuekkito
ICKT: Inchinkoto
JDI: Jidabokuippo
KBG: Keishibukuryogan
KHT: Kihito
NYT: Ninjinyoeito
OGT: Orengedokuto
RKT: Rikkunshito
SBT: Saibokuto
SHT: Seihaito
YKS: Yokukansan.
